# The effect of 5α-oleandrin on keloid fibroblast activities

**DOI:** 10.1186/s12919-019-0177-6

**Published:** 2019-12-16

**Authors:** Ishandono Dachlan, Yohanes Widodo Wirohadidjojo, Mae Sri Hartati Wahyuningsih, Teguh Aryandono, Hardyanto Soebono, Dwiki Afandy

**Affiliations:** 1 Plastic and Reconstructive Surgery Division, Department of Surgery, Faculty of Medicine, Public Health and Nursing, Universitas Gadjah Mada, Dr. Sardjito Hospital, Jl. Kesehatan No. 1, North Sekip, Yogyakarta, 55281 Indonesia; 2 Department of Dermato-venereology, Faculty of Medicine, Public Health and Nursing, Universitas Gadjah Mada/ Sardjito-Hospital, North Sekip, Yogyakarta, Indonesia; 3 Department of Pharmacology and Therapy, Faculty of Medicine, Universitas Gadjah Mada, North Sekip, Yogyakarta, Indonesia; 4 Oncology Surgery Division, Department of Surgery, Faculty of Medicine, Public Health and Nursing, Universitas Gadjah Mada/ Sardjito-Hospital, North Sekip, Yogyakarta, Indonesia; 5 Department of Dermato-venereology, Faculty of Medicine, Public Health and Nursing, Universitas Gadjah Mada/ Sardjito-Hospital, North Sekip, Yogyakarta, Indonesia; 6 Faculty of Medicine, Public Health and Nursing, Universitas Gadjah Mada/ Sardjito-Hospital, North Sekip, Yogyakarta, Indonesia

**Keywords:** Cell proliferation, Fibroblasts, Keloid, Mitomycin, 5α-oleandrin

## Abstract

**Background:**

Keloids develop due to hyperactivity of keloid fibroblast (KF) in proliferation, migration, and collagen deposition along with low rates of collagen degradation. These are a result of the Wnt/β catenin signaling pathways under stimulation of TGF-β. 5α-oleandrin can suppress Wnt-targeted genes of osteosarcoma cells. We aimed to evaluate the anti-fibrotic effects of 5α-oleandrin on KF activities.

**Methods:**

We collected the core of keloid materials from six patients who underwent keloid debulking surgery. Passage 4 of KF cells were then treated with mitomycin-C, 5α-oleandrin, and dilution medium as the negative control. To determine the effective dose of 5α-oleandrin, we diluted 5α-oleandrin into various concentrations. The incubation periods were 24 h, 48 h, and 72 h. The anti-proliferation and anti-fibrotic properties were measured using standard assay.

**Results:**

Both the mitomycin-C and 5α-oleandrin treated groups indicated decrease in proliferation index (86.16 ± 4.20% and 73.76 ± 4.94%, respectively), collagen deposition index (90.26 ± 1.72% and 71.35 ± 4.26%, respectively), and migration capacity (33.51 ± 1.50% and 28.57 ± 1.58%, respectively). These were significant changes (*p* ≤ 0.05) compared to the non-treated group. Antifibrotic activities of 5α-oleandrin in cellular proliferation and collagen deposition were better than mitomycin-C.

**Conclusions:**

The 5α-oleandrin has good antifibrotic effect in keloid fibroblast activities.

## Background

Keloids are a fibroproliferative benign tumor that only affects human skin with characteristic overgrowth of scar tissues that exceeds the original wound size [1]. This characteristic is caused by keloid fibroblast (KF) proliferation and abundant production of collagen [2–4] together with the low activity of matrix metalloproteinase (MMP) on extracellular matrix degradation [5, 6]. Various keloid treatments starting with surgical procedures to intralesional corticosteroids and various anti-cancer agents such as bleomycin [7], mitomycin [8], and fluorouracil [9], (or a combination of these) have been performed [10], but the results are unsatisfactory and they still reveal a high recurrence rate.

Each transforming growth factor beta (TGF-β) subclass has a different role in wound healing, and transforming growth factor beta 1 (TGF-β1) is well known as an important growth factor in keloid formation [10]. This growth factor can induce Wnt/β catenin signaling pathways whereas overexpression of Wnt is parallel to collagen deposition in keloid tissues [11] as well as collagen production in KF [12, 13]. The Wnt/β catenin signaling pathway is not only responsible for collagen production in keloid fibroblasts, but it also has an important role in inducing transformation of human dermal microvascular endothelial cells to become KF [13].

Recently, many natural products are tested for anticancer properties including oleandrin (C_32_H_48_O_9_) isolated from *Nerium indicum* Mill. This material can significantly inhibit cellular proliferation and cellular invasion by suppressing Wnt-targeted genes of osteosarcoma cells [14]. It also has a cytotoxic effect against HeLa cells with an IC50 of 8.38 × 10^− 6^ mM, but it is less cytotoxic against normal human cells [15]. One study showed that the administration of *Nerium oleander* taken orally for 21–28 days is well tolerated in heavily pretreated patients with advanced solid tumors [16]. Therefore, our study evaluated the anti-proliferation and anti-fibrotic properties of oleandrin on keloid fibroblast cultures.

## Materials and methods

This study conformed to the ethical principles outlined in the Declaration of Helsinki and received approval from our Institutional Review Board (#KE/FK/83/EC/2013).

### Isolation and culture of keloid fibroblast

We collected the core of keloid materials from six patients who underwent keloid debulking surgery after completing informed written consent forms. The age range of the patients was 18–23 years old. A 2 cm^3^ core of each material was thinly sliced into 0.2–0.3 cm^3^ pieces and cultured via an explant method in Dulbecco’s Modified Eagle’s Medium (DMEM, Gibco®, USA) containing 10% fetal bovine serum (FBS, Gibco®, USA) and 1% penicillin/streptomycin (Gibco®, USA) at 37 °C and 5% CO_2_. The spindle-shaped cells that grew out from the explants then were sub-cultured until passage-4.

### Experiments

Passage 4 of KF cells from each patient were then treated with mitomycin-C, 5α-oleandrin, and dilution medium as the negative control. We used 5α-oleandrin purchased from the Department of Pharmacology and Therapy, Faculty of Medicine, our university as the experimental groups; mitomycin-C (Kyowa, Tokyo, Japan) was the positive control. Both materials were diluted in DMEM containing 10% fetal bovine serum plus 1% penicillin/streptomycin in various concentrations. We used the lowest effective dose of mitomycin-C (30 μM) as previously reported by Dachlan et al. [17] as the positive control. To determine the effective dose of 5α-oleandrin, we diluted 5α-oleandrin into various concentrations, and the highest dose was considered to be half of the dose of mitomycin-C. The incubation periods were 24 h, 48 h, and 72 h***.***

### Measurement of variables

#### Proliferative index

The anti-proliferation property was measured by measuring cellular viabilities using MTT (3-(4,5-dimethylthiazol-2-yl)-2,5-diphenyltetrazolium bromide) purchased from MP Biomedicals, France. The resulting optical density (OD) of the formazan product produced by the MTT and living cells was measured at 570 nm. The OD of the non-treated group was defined as 100% proliferation, and the proliferation index of various treated groups was counted as: (OD of treated group **/** OD of paired non-treated group) × 100%.

#### Collagen deposition

The anti-fibrotic property was quantitated by measuring the collagen deposition using insoluble collagen of Sirius red (purchased from Sigma-Aldrich, Steinheim, Germany) assay based on the method of Taskiran et al. [18]. The OD of the Sirius red-bound collagen represented the amount of insoluble collagen. This was read at 570 nm. The OD of the non-treated group was defined as 100% of the ability to deposit collagen. The ability of the various treated groups was counted as (OD of treated group / OD of paired non-treated group) × 100%.

#### Cellular migration

The anti-fibrotic property was also determined via a cellular migration assay based on Liang et al. [19]. Briefly, after serum starvation, the bottoms of the wells were linearly scratched with the blunt tip of a 32G sterile needle through the center of the well bottom. After cultivation with various media and incubations, the cells were then stained with Meyer’s hematoxylin and microscopic photo images were taken using a Moticam 350 (China) camera in JPG format. The scratch line was measured via the blue (fibroblasts) and white pixels (empty space). The migration rate was determined via (blue color pixel of KFs along scratch line / total pixels along the scratch line) × 100%. Migration capacity of the treated group was counted as: migration rate of the treated group divided by migration rate of the paired control group.

### Statistical analysis

All data were presented as a mean ± standard error. We used Analysis of Variance (ANOVA) followed by Fisher’s Least Significant Difference (LSD) to analyze data with normal distribution. For data with abnormal distribution, we used the Friedman followed with Wilcoxon rank sign test. *p* < 0.05 was considered as significant level.

## Results

Both the mitomycin-C treated group and the 5α-oleandrin-treated group indicated a decrease in proliferation (86.16 ± 4.20% and 73.76 ± 4.94%, respectively), collagen deposition index (90.26 ± 1.72% and 71.35 ± 4.26%, respectively), and migration capacity (33.51 ± 1.50% and 28.57 ± 1.58%, respectively). This was a significant change (*p* ≤ 0.05) compared to the non-treated group. The comparison between mitomycin C and 5α-oleandrin proliferation indices, collagen deposition, and migration capacity are detailed below.

**Fig. 1:**
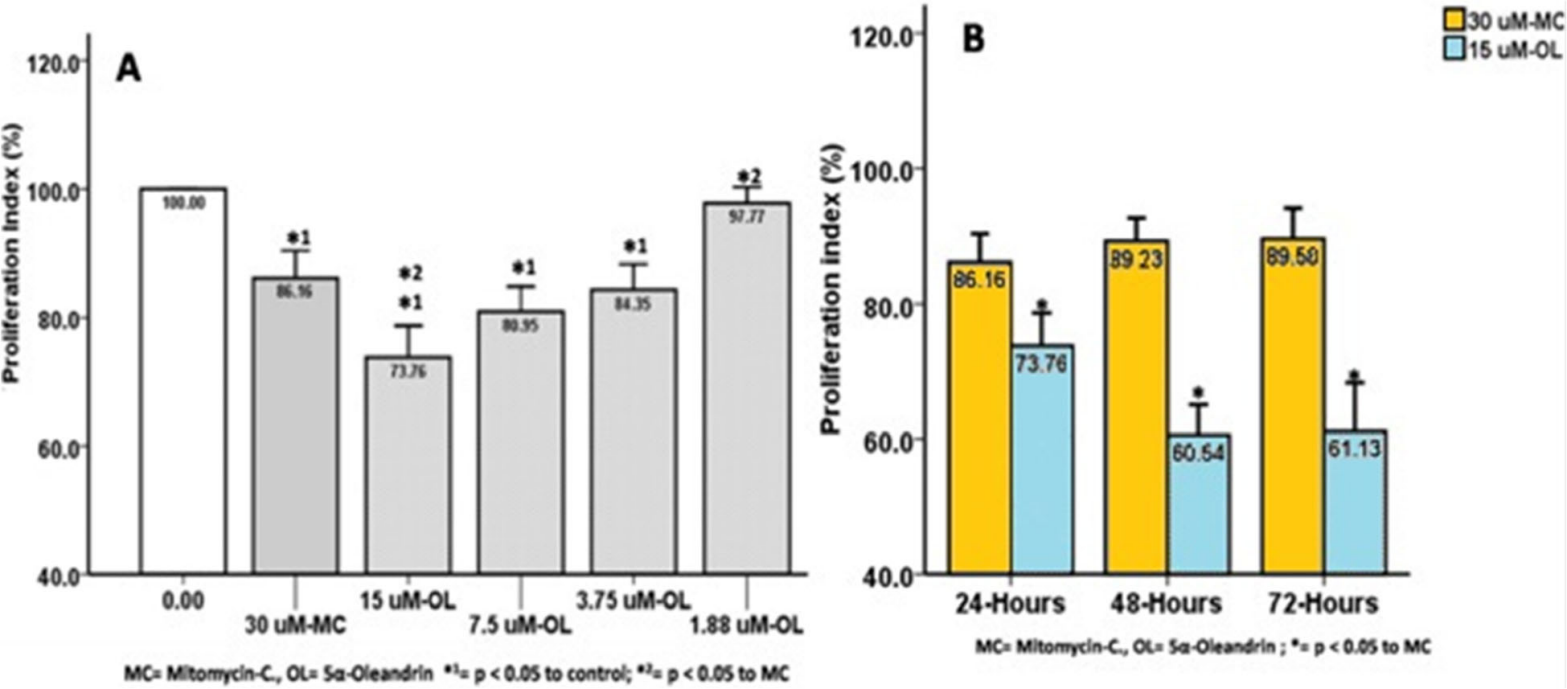
Suppression of the proliferation index of KF by mitomycin C and 5α-oleandrin: **a** in serial dilutions in 24 h of incubation, and **b** in various incubation periods

5α-oleandrin suppresses KF proliferation at ≥3.75 uM; the 15 uM 5α-oleandrin was better than mitomycin C (Fig. 1a). This suppression effect persisted until 72 h of incubation period (Fig. 1b).

**Fig. 2:**
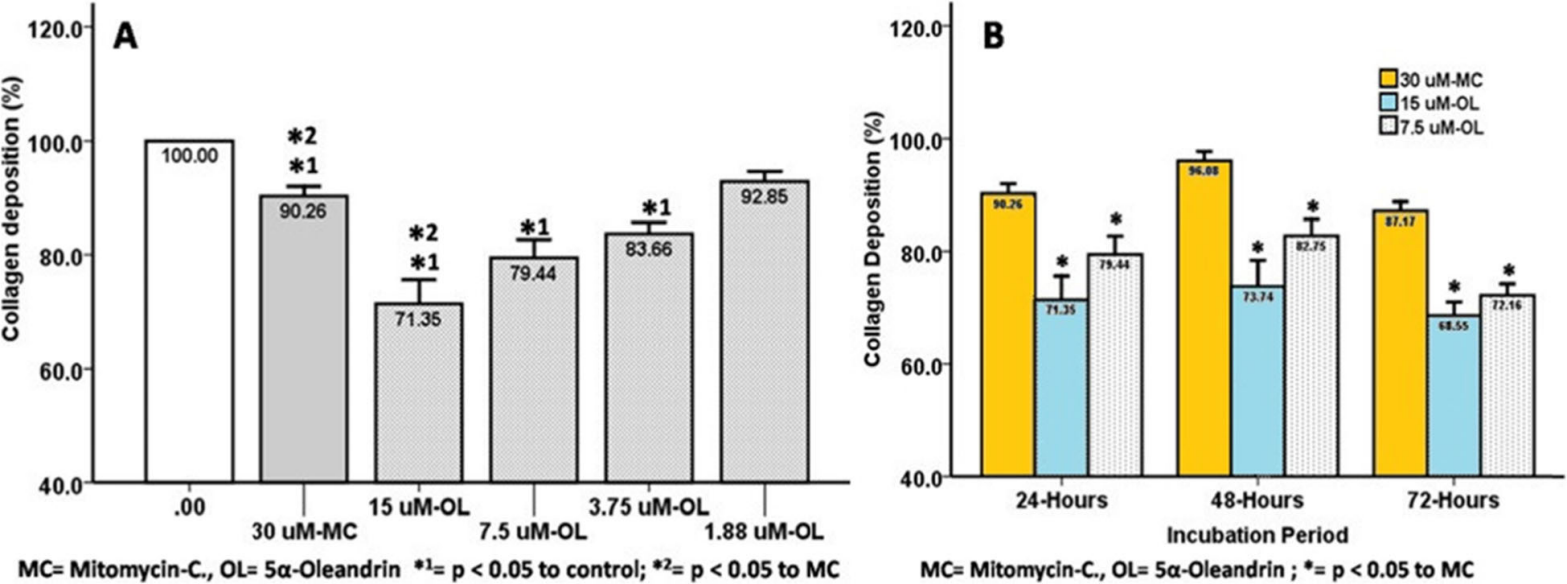
Suppression of the collagen deposition by mitomycin C and 5α-oleandrin: **a** in serial dilutions, and **b** in various incubation periods

5α-oleandrin suppresses KF in collagen deposition at ≥3.75 uM; 7.5 uM 5α-oleandrin was better than mitomycin C (Fig. 2a). This suppression effect persisted until 72 h of incubation period (Fig. 2b).

**Fig. 3:**
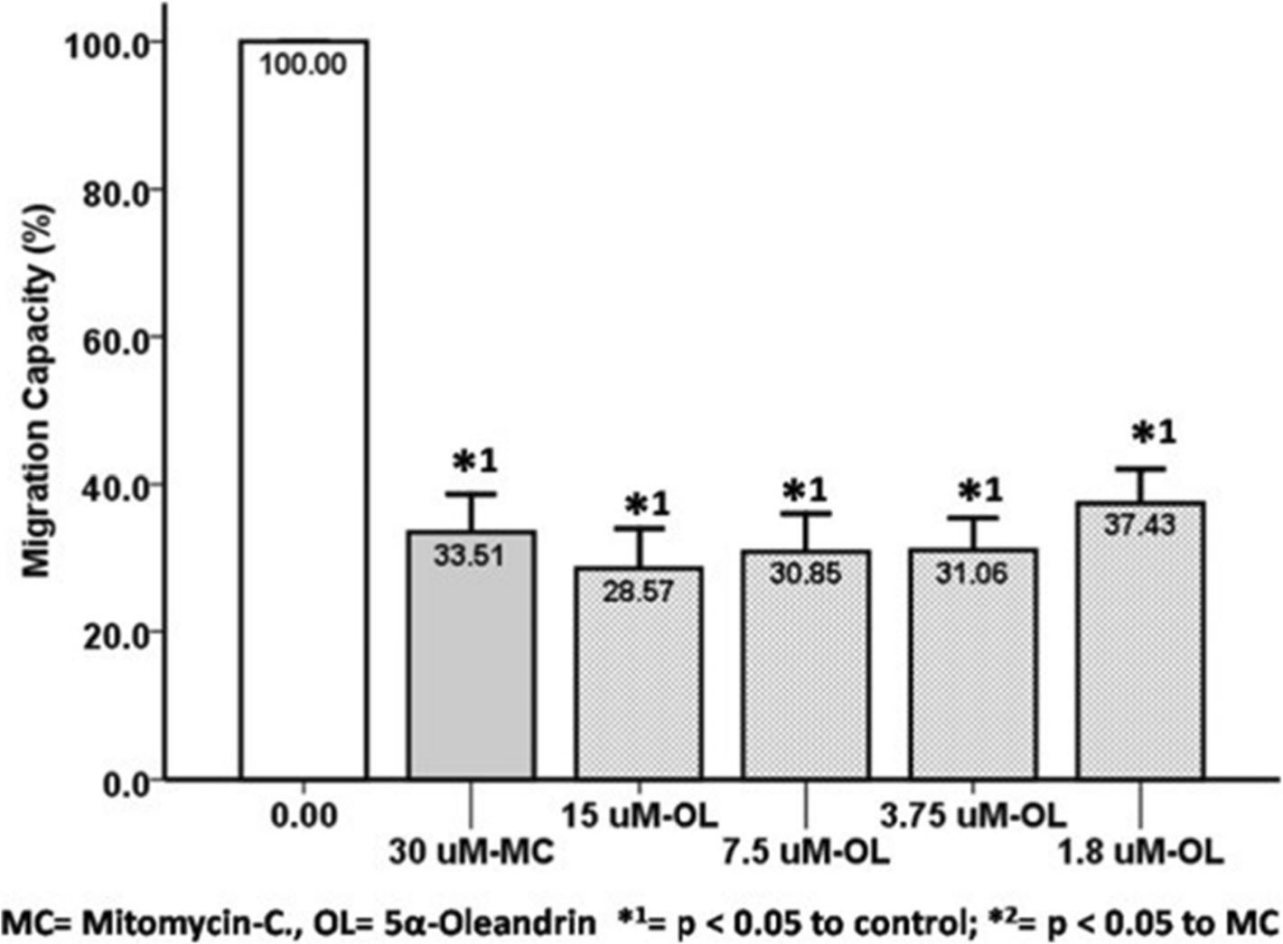
Suppression of the migration capacity of KF by mitomycin C and 5α-oleandrin

In addition, 5α-oleandrin also suppresses KF migration at all dilution levels; none of these were better than 30 uM mitomycin-C (Fig. 3).

**Fig. 4:**
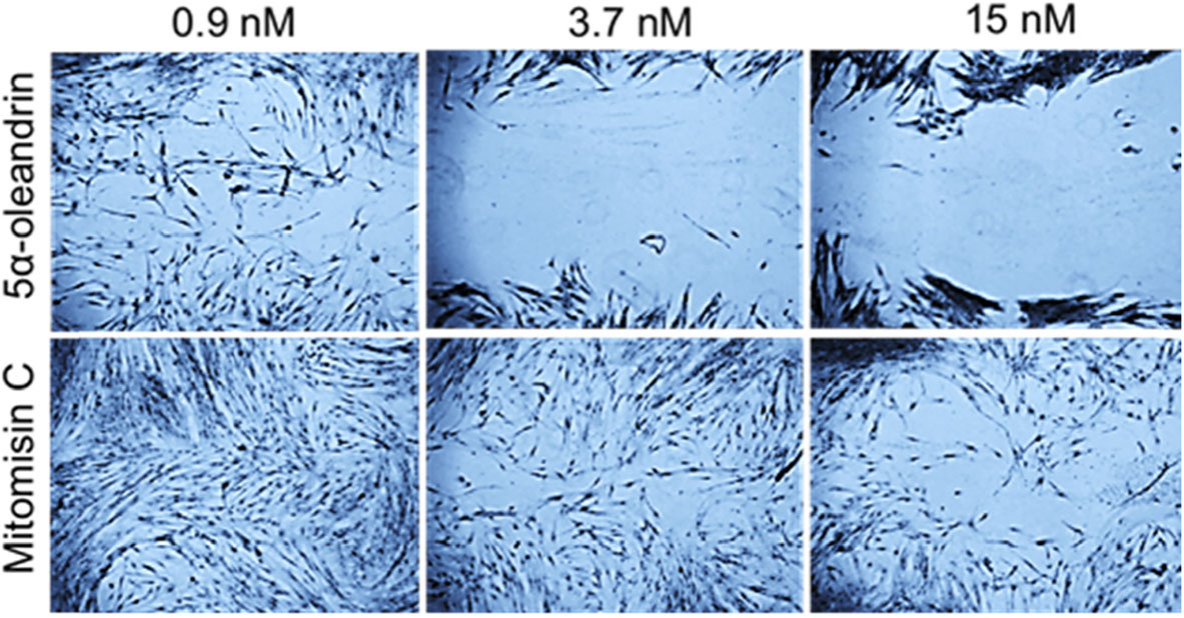
The comparison between the effect of 5α-oleandrin and mitomycin C on keloid fibroblast migration activity after 72 h

The activity of keloid fibroblast migration after 72 h was higher in the administration of 5α-oleandrin than that of mitomycin C (*P* < 0.05) as shown in Fig. 4.

## Discussion

Until recently, representative animal models for studies of keloids have not been available. Therefore, most experiments searching for keloid therapeutics use keloid fibroblasts. For example, Richard et al. [20] indicated that mitomycin-C could inhibit KF proliferation by suppressing DNA synthesis. Dachlan et al. [17] showed that 30 uM mitomycin-C could suppress keloid fibroblast proliferation and collagen synthesis. Both studies are based on Stewart et al. [8] who used topical application of mitomycin-C for clinical wound repair trials to prevent keloid. Mitomycin-C influences DNA synthesis. Oleandrin is a new cytotoxic agent and suppresses Wnt-targeted genes [14] to indirectly affect collagen deposition in keloid tissues [11] and collagen production of keloid fibroblasts [12, 13]. The Wnt/β catenin signaling pathway is induced by TGF-β1 as an important growth factor in the keloid pathological mechanism. Chua et al. [21] reported that the canonical Wnt/β-catenin signaling is involved in keloid pathogenesis. Ma et al. [14] demonstrated that oleandrin could reduce the nuclear β-catenin which is consistent with reports that the suppression of the Wnt/β-catenin signaling pathway would lead to the reduction of nuclear β-catenin [22]. Oleandrin had a remarkable inhibiting effect on the downstream molecules of the Wnt/β-catenin signaling pathway through downregulation of the mRNA levels of c-myc, survivin, cyclin D1, MMP- 2 and MMP-9 [14]. In our experiment, neither Wnt nor β catenin expression were measured. Our experiment reveals that 5α-oleandrin at a half dose of mitomycin-C can suppress keloid fibroblast proliferation better than mitomycin-C after 24 h of incubation. It has persistent activity up to 72 h (Fig. 1). Similar results were also found in collagen deposition even at 25% of the dose of mitomycin-C (Fig. 2). The 5α-oleandrin can suppress keloid fibroblast migration similar to mitomycin-C even at the lowest dilution level (Fig. 3). In addition, future study using animal model is necessary to clarify and confirm our findings.

In clinical practice, drugs for keloid can reduce keloid tension and size. Both variables are responsible for keloid fibroblast activities including the proliferation and deposition of collagen material [2, 3] plus migration to invade the normal adjacent skin [14]. In tandem with keloid surgery, keloid drugs can prevent recurrent keloids by suppressing residual keloid fibroblasts on the margin of the wound to proliferate and deposit collagen. Similar results are achieved with 5α-oleandrin. Topical delivery of 5α-oleandrin is possible based on the physicochemical properties of 5α-oleandrin including its low molecular weight (576.72 Da) and lipid solubility [23]. Although irritant contact dermatitis caused by cutaneous exposure of oleander leaves has been reported [24], the allergenic properties have not been adequately studied. Generally, no positive patch test can be obtained [25]. So, Clinical trials of this material either as a single therapy versus a topical standardized drug or as adjunctive treatment of keloid surgical procedures are possible.

It should be noted that we only performed MTT assay in this study. It was not ideal one for measurement of proliferation indices. In addition, we did not use the untreated cells as a control for For the proliferation index and collagen deposition during the incubation period. Due to limitation of resources, we could not perform the proliferation indices test, such as 5-bromo-2′-deoxyuridine (BrdU-) or 5-ethynyl-2′-deoxyuridine (EdU-)incorporation assay, and the cell death measurement test, such as TUNEL or anti-caspase-3 staining, becoming a limitation of our study.

## Conclusions

5α-oleandrin has good anti-fibrotic effect. Further study is still necessary to reveal more specific manner of this substance as anti-proliferation agent. BrdU- or EdU- incorporation assay are suggested method to examine cells proliferation. Either BrdU and EdU assay are sensitive method but special precautions have to be noted due to their toxic, potential mutagenic, and/or teratogenic effects.

## Data Availability

All data generated or analyzed during this study are included in the submission. The raw data are available from the corresponding author on reasonable request.

## Abbreviations

ANOVA: Analysis of variance
DMEM: Dulbecco’s modified eagle’s medium
EdU: 5-ethynyl-2′-deoxyuridine
FBS: Fetal bovine serum
KF: Keloid fibroblast
LSD: Least significant diffrenece
MMP: Matrix metalloproteinase
MTT: 3-(4,5-dimethylthiazol-2-yl)-2,5-diphenyltetrazolium bromide
OD: Optical density
TGF-β: transforming growth factor bet
